# How Behavioral Changes Can Indicate Serious Cerebral Pathology: A Case Report of Concomitant Olfactory Neuroblastoma and Distemper Virus Encephalitis in a Swiss Shepherd Dog

**DOI:** 10.3390/vetsci4030042

**Published:** 2017-08-28

**Authors:** Dario Candini, Ilaria Biasato, Paulo Ricardo Dell’Armelina Rocha, Elena Grego, Maria Teresa Capucchio, Cristina Vercelli

**Affiliations:** 1 Veterinary practitioner, 10144 Turin, Italy; gufo000@gmail.com; 2 Department of Veterinary Science, University of Turin, 10095 Grugliasco, Italy; ilaria.biasato@unito.it (I.B.); elena.grego@unito.it (E.G.); mariateresa.capucchio@unito.it (M.T.C.); 3 Graduate Program in Environmental and Experimental Pathology, Paulista University, (UNIP), São Paulo 04313-210, Brazil; ricardodellarmelina@gmail.com

**Keywords:** brain, canine distemper virus, dog, immunohistochemistry, olfactory neuroblastoma

## Abstract

Behavioral alterations in dogs are not easy to understand and cure. The situation is more difficult when an encephalitis due to *Canine Distemper Virus* (CDV) and a concomitant olfactory neuroblastoma are present. This case report deals with the story of a 5-year-old Swiss shepherd dog with behavioral changes, seizures, epistaxis and ataxia. Following clinical and laboratory exams, a suspected diagnosis of CDV infection was hypothesized, and a therapy based on Ω-interferon was administered. Every supporting therapy failed and the worsening of the clinical conditions led to the euthanasia of the patient. A neoformation in the right frontal lobe was found post mortem. Histopathology and immunohistochemistry investigation showed a non-suppurative demyelinating encephalitis, suggestive of CDV infection, and a desmoplastic epithelioid olfactory neuroblastoma. To the best of authors’ knowledge, this is the first clinical pathological report of a non-suppurative encephalitis due to CDV infection and olfactory neuroblastoma in a dog.

## 1. Introduction

Dogs are nowadays considered family members, and consequently, when pets start to display undesirable behavior, the relationship could be damaged. It is hard to understand the reasons for destructiveness, fear, anxiety, and even aggressiveness, and the evaluation can be difficult, both for owners and veterinarians; some tools, like questionnaires for owners, educational training and clinical examination, can be performed to understand the causes of the behavioral alteration and to try to find a solution. However, there are some cases that are not only related to psychological troubles, but that demonstrate systemic or neurologic pathologies. 

## 2. Case History 

Our report deals with the case of a 5-year-old male Swiss shepherd dog that lived in Turin (Italy). The dog began to demonstrate behavioral abnormalities, like a tendency to stay alone, lack of sociability with other dogs, and aggressiveness towards owners. The first line intervention was educational training with a canine educator, who tried to manage the patient and, in the absence of results, recommended a visit to a veterinarian behaviorist; treatment with fluoxetine (1 mg/kg orally once a day) and then, in the absence of results, with clomipramine hydrochloride (2 mg/kg orally once a day), was administered. The behavioral status remained unchanged even with drug administration for another 6 months, but suddenly the patient was referred for emergency treatment to clinicians (Dario Candini and Cristina Vercelli) for a seizure, which was controlled with a single administration of diazepam (0.2 mg/kg intravenously (IV)) and phenobarbital (3 mg/kg IV). When the emergency was solved, the clinicians spoke with owners to collect anamnestic information, and when the condition of the patient was stable, a complete physical examination was performed. The dog demonstrated ataxic gait, tremors leading to ataxia, right nostril epistaxis and ipsilateral eye lachrymation, congested mucosae, body temperature at 38.9 °C, and no alteration at thoracic auscultation and abdomen examination. Anamnesis recorded regular vaccination until the age of 3.5 years for Canine Distemper Virus (CDV-Onderstepoort strain), Canine Adenovirus (Manhattan LPV3 strain), Canine Parvovirus (Intervet 154 strain), Parainfluenza virus, and Leptospirosis (*Leptospira interrogans* serovars *canicola* and *icterohaemorrhagiae*). Prophylaxis against filariosis, leishmaniosis and ectoparasites was administered regularly. 

The following differential diagnoses were hypothesized: idiopathic epilepsy, infective or parasitic disease causing encephalitis (i.e., toxoplasmosis, Neospora caninum, distemper virus, erlichiosis, rickettsiosis) central nervous system neoplasia or metabolic syndrome (i.e., hypoglicemia, secondary hepatic encephalophaty due to a shunt, hypo/hyperadrenocortisism) [1]. Blood analysis showed no significant alterations, other than a decrease of creatinekinase (CK) and total protein levels. Serum protein electrophoresis revealed a decrease of α1 and β1 globulins, and an increase of α2 and β2 fractions. Immunenzymatic rapid test to detect antigens of CDV (Quick test-Whitness) resulted positive, while rapid tests for toxoplasmosis, filariosis, and leishmaniosis resulted negative. X-ray of the head showed intense radiopacity involving the right nostril, without bone alteration. Owners did not accept performance of a magnetic resonance imaging (MRI) scan, but permitted the collection of cerebrospinal fluid (CSF). Cytology revealed moderate mononuclear pleocytosis (75% little lymphocytes, 20% neutrophils and 5% macrophages), indicating a nonsuppurative encephalitis. One-step real time PCR (Qiagen company, Hilden, Germany) on nucleic acid extracted from CSF, amplifying a 335bp fragment using a first primer of 5′ GATAAAGCATGTCATTATAGTCCTAA 3′ and second primer of 5′ CTTGAGCTTTCGACCCTTC 3′ (Trizol, Life Technologies, Thermo Fisher Scientific, Waltham, MA, USA), demonstrated CDV in CSF. A nasal wash was performed, and cytological analysis showed neutrophils including coccobacilli, along with squamous metaplasia and strong dysplasia of the epithelial cells, concordant to a mucopurulent rhinitis, coherent with CDV nasal epithelium colonization [2].

The suspected diagnosis was CDV.

Administration of phenobarbital (2.5 mg/kg orally twice a day) was always maintained, and feline recombinant Ω-interferon (VirbagenOmega, Virbac—2.5 UM/kg IV for 3 consecutive days) was started. The antiviral effectiveness of Ω-interferon against CDV was established to be four times higher than α-interferon against CDV [3]. The therapy was repeated after 4 weeks. During the following three months, serial clinical examinations and determination of barbituremia were performed; no improvements of the clinical conditions were seen, and every supporting therapy failed. During the last two weeks, the dog was anorexic, ataxic, and became blind.

Veterinarians and owners agreed to proceed with the euthanasia, and the dog was submitted to post mortem examination. 

**Figure 1:**
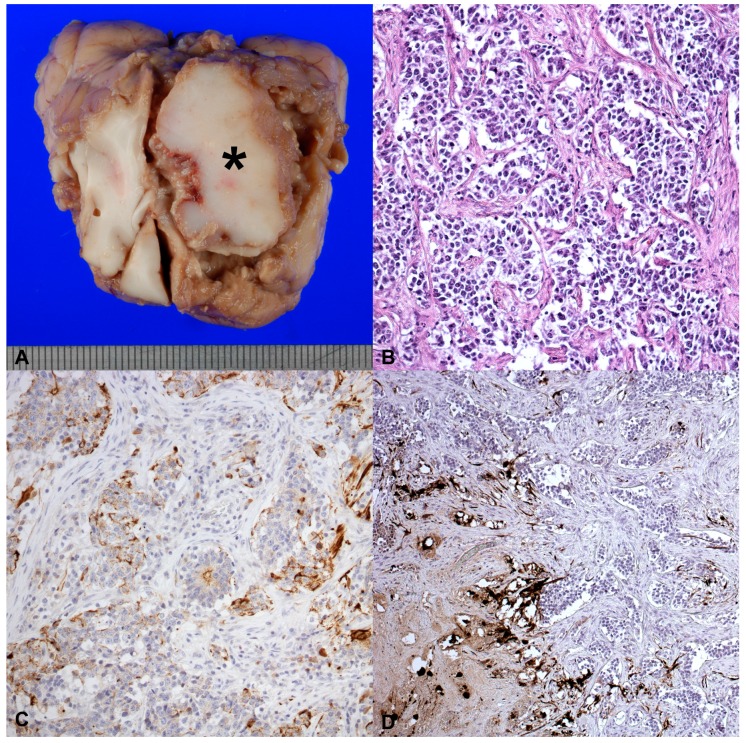
(**A**) Macroscopic aspect of tumor that was found in the right frontal sinus of a 5-year-old male Swiss shepherd dog, along with a wide, irregular, brown-white and firm lesion, measuring 3.5 × 3 × 3 cm, occupying the right frontal lobe and compressing the contiguous cerebral parenchyma; (**B**) Round-to-oval cells organized in sheets or pseudorosettes supported by hypocellular zones of delicate fibrillary stroma; 40×; (**C**) Neoplastic cells densely organized. The picture shows the positivity for citokeratines, 20×; (**D**) The picture shows tumor cells isolated or small cluster, that were immunopositive for NF; 20×.

External body examination did not show significant alterations, except for the slimming. The brain was collected and gross examination of nasal cavity shown a stringy exudate in the right frontal sinus, along with a wide, irregular, brown-white and firm lesion, measuring 3.5 × 3 × 3 cm, occupying the right frontal lobe and compressing the contiguous cerebral parenchyma (Figure 1A). 

Brain, thoracic and abdominal organs were fixed in a 10% buffered formalin solution and routinely embedded in paraffin wax blocks. Subsequently, 4 µm tissue sections were cut for histopathologic and immunohistochemical (IHC) evalutations.

Microscopically, no significant histological abnormalities were detected in the thoracic and abdominal organs. The brain lesion was unencapsulated, infiltrative and composed by markedly pleomorphic, round-to-oval cells (with round- to oval-shaped nuclei, dispersed chromatin and prominent nucleoli), sometimes densely organized in nests or pseudorosettes supported by hypocellular zones of fibrillary stroma (Figure 1B). A total of 3–4 mitosis per high-power field (HPF, 40×) were counted. Multifocal and large areas of necrosis, hemorrhages and perivascular lymphocytic cuffing were also observed. Selected samples of the neoformation were specifically immunolabeled (Table 1). 

Densely organized neoplastic cells were negative for Glial fibrillary acidic protein (GFAP), vimentin and S-100, while isolated or small cluster tumor cells were intensely immunopositive for Neuron enolase (NSE), Neurofilament (NF), and cytokeratins (Figure 1C,D). Stroma demonstrated moderate to severe immunopositivity for vimentin, GFAP and S-100. The Ki-67 proliferation index ranged from 47.75% to 75.5%. Histopathological and immunohistochemical findings led to the diagnosis of olfactory neuroblastoma (ONB). Multifocal and moderate vacuolization of the white matter indicating demyelination, associated with the presence of some vacuolizations, glial cells, small vessel proliferation and a mild non suppurative inflammation, was observed in the cerebral cortex, mesencephalon and pons. Multifocal and mild leptomeningitis with perivascular cuffing, mostly composed by lymphocytes, plasma cells and macrophages were observed in all brain sections. Cerebrum, pons and mesencephalon sections were immunolabeled using a D110, a specific monoclonal antibody for CDV (positive control: cerebellum of a puppy dead for CDV), according to the method described by Bollo et al. [4], but resulted negative. 

Based on the gross, microscopical and immunohistochemical results, and according to Beineke et al. [5], the neurological signs of the dog of this report were caused by a concomitant distemper encephalitis and an olfactory neuroblastoma. 

## 3. Discussion

In the present case report, the identification of neurological symptoms at clinical examination was partially suggestive of CDV infection. Indeed, the diagnosis was confirmed only by cytological analysis and real-time PCR (RT-PCR) of the CSF. At post mortem examination, moderate and disseminated demyelination associated with the presence of axonal degeneration, glial proliferation and nonsuppurative inflammation were observed as the most relevant brain lesions. The identification of demyelinating encephalomyelitis in white matter is the main manifestation of CDV, and is in agreement with previous reports [5,6,7]. The present case report demonstrated a moderate and disseminated demyelination associated with the presence of axonal degeneration and glial proliferation. These demyelinated areas could be responsible for seizures and also for behavioral alterations [8]. On the other hand, intracranial neoplasia could also cause the same symptoms, and might also be responsible for epistaxis, sneezing and nasal discharge, which might be suggestive of tumors [9]. 

The lack of positivity of IHC for D110 could be explained by the administration of feline recombinant Ω-interferon, which is characterized by high antiviral activity [3]. The hypothesis of an atypical CDV infection, as reported by Galán et al. [10], was taken into account. However, the presence of an inflammatory reaction may indicate that a slow viral infection rather than a totally masked or latent virus could be involved. Another form of non-purulent encephalitis, old dog encephalitis (ODE), which is an extremely rare manifestation of CDV-encephalitis and is characterized by progressive cortical deterioration due to chronic multifocal lymphoplasmacytic encephalitis with extensive perivascular cuffing and astrocytosis, was also considered [11]. ODE usually occurs in dogs older than 6 years, most frequently with a complete vaccination history [12]. Considering the rapid evolution of the neurological symptoms and the identification of different histopathological findings in the dog of the present case report, an ODE diagnosis was excluded.

The post mortem examination allowed the identification of a concomitant olfactory neuroblastoma (ONB), which is a rare and malignant neuroectodermal tumor that derives from olfactory neuroepithelium, arises within the upper nasal cavity at the level of the cribriform plate [13], and may extend to the paranasal cavity and intracranial cavities [14]. The common clinical presentation of nasal tumors that extend into the intracranial cavity includes exhibiting seizures and abnormal behavior, rather than signs suggestive of upper respiratory disease [14]. The knowledge about the correlation between behavioral changes and the presence of intracranial tumors is very poor in veterinary medicine. Some physicians have investigated the correlation between human personality changes and psychiatric disturbances following brain tumors, which could be mainly due to the putative role of organic mechanisms such as location of the lesion, or effects of surgical treatment [15].

The histopathological features of tumor cells observed in the dog of the present case report are consistent with those reported in previous studies about ONBs [13]. Furthermore, the presence of high mitotic activity (demonstrated by the number of mitosis per HPF and the Ki-67 proliferation index), marked cell pleomorphism and extended necrosis suggested the diagnosis of a less differentiated histotype and indicated the malignancy of the tumor. 

It was demonstrated that a correlation between the presence of virus and tumors in animals exists; some examples include pulmonary adenocarcinoma in sheep caused by a retrovirus, and species-specific oncogenes in rodents, fowl, feline and fish [16]. Moreover, early-age exposure (fetal or neonatal age) can cause permanent alterations of the immune system that could possibly induce tumor formation [16]. Nevertheless, a direct correlation between the CDV and the onset of ONB has not been previously assessed. On the contrary, it has been reported that some viruses (e.g., rabies) could be used to trace neuronal cells [17], and also that several attenuated Newcastle Disease Virus (NDV) strains were evaluated in pre-clinical studies in murine xenograft models where an efficacy against several human cancers such as colon, large cell lung, breast, prostate carcinomas, and neuroblastoma demonstrated a regression upon NDV treatment [18].

Taking into account all this information, in the authors’ opinion, it is not possible either to exclude a prodromic effect of the CDV regarding ONB, or the limitation of the expansion of the tumor due to the simultaneous presence of the distemper virus. 

## 4. Conclusions

In the present paper, the authors reported the case of a 5 years old Swiss shepherd dog that, at the beginning, showed behavioral changes and epilepsy, followed by an *intra vitam* diagnosis of CDV. The post mortem investigation demonstrated a concomitant olfactory neuroblastoma and a demyelinating encephalitis. 

To the best of the authors’ knowledge, this is the first clinicopathological description of the simultaneous presence of ONB and CDV brain infection in a dog.

## Figures and Tables

**Table 1:** Antibodies and their dilution used to perform immunohistochemistry on the brain mass.

Antibody	Type/Company	Dilution	Tumour	Stroma
Vimentin	Monoclonal Mouse, Anti-Vimentin, Clone V9, Dako, Glostrup, Denmark	1:200	−	+
Cytokeratins	Monoclonal Mouse, Anti-Human Cytokeratins, Clones AE1/AE3, Dako, Glostrup, Denmark	1:50	+	−
Glial fibrillary acidic protein (GFAP)	Polyclonal Rabbit, Anti-Glial Fibrillary Acidic Protein, Dako, Glostrup, Denmark	1:5000	−	+
S-100	Polyclonal Rabbit, Anti-Cow S-100, Z0311, Dako, Glostrup, Denmark	1:200	−	+
Neuron enolase (NSE)	Monoclonal Mouse, Anti-Human Neuron Specific Enolase, Clone BBS/NC/VI-H14, Dako, Glostrup, Denmark	1:500	+	−
Neurofilament (NF)	Monoclonal Mouse, Anti-Human Neurofilament Protein, Clone 2F11, Dako, Glostrup, Denmark	1:10,000	+	−
